# Communication, the Heart of a Relationship: Examining Capitalization, Accommodation, and Self-Construal on Relationship Satisfaction

**DOI:** 10.3389/fpsyg.2021.767908

**Published:** 2021-12-13

**Authors:** Priscilla Maria De Netto, Kia Fatt Quek, Karen Jennifer Golden

**Affiliations:** ^1^Department of Psychology, Jeffrey Cheah School of Medicine and Health Sciences, Monash University Malaysia, Subang Jaya, Malaysia; ^2^Jeffrey Cheah School of Medicine and Health Sciences, Monash University Malaysia, Subang Jaya, Malaysia

**Keywords:** communication, capitalization, accommodation, positive psychology, dating, satisfaction, Malaysia, culture

## Abstract

The study of processes that enrich positive relationships has been an under-researched area within positive psychology practice. The way an individual responds during couple conflicts (accommodation response) and toward the disclosure of good news of a partner (capitalization response) has been linked to relationship quality. Although the accommodation and capitalization communication processes are part and parcel of our everyday lives, the two processes have been examined separately and dominated by the Western perspectives in past research. Prior work has suggested that Western and Asian cultures differ in expressing and perceiving beneficial communication behaviors. Yet, it is still unclear which accommodation and capitalization responses matter the most from an Asian lens. To date, there is no research examining these interconnected variables simultaneously in Asia, specifically in Malaysia. In this study, two forms of communication processes, namely, (1) accommodation and (2) capitalization, were explored concurrently to disentangle the unique associations and influence on relationship satisfaction. This study also sought to understand the moderating effects of culture in terms of interdependent self-construal on the link between these two communication processes and relationship satisfaction. Responses of 139 Malaysians in dating relationships between the age of 18 and 30 years (*M*_age_ = 23.15) were collected through online surveys. An active and constructive reaction was captured as the most favorable response through both the capitalization and accommodation processes. Prominently, an active-constructive capitalization response bore the strongest influence on relationship satisfaction above and beyond other responses. A passive and constructive response was revealed only fruitful for disclosures of positive news and not during conflicts. Conversely, in the destructive paradigm, passive-destructive responses were the most detrimental factor in relationships compared to other destructive responses. The results also uncovered that interdependent self-construal did not moderate the two forms of communication processes. However, the findings discovered unexpected individual and cultural variations. This pioneering study is a noteworthy addition to the positive psychology literature from an Asian standpoint. It highlights the significance of not only protecting relationships through better conflict management but also enriching relationships by capitalizing on the positive aspects across the lives of the couple, ultimately providing a greater holistic insight into cultivating flourishing lives.

## Introduction

“*Man is by nature a social animal … Anyone who either cannot lead the common life or is so self-sufficient as not to need to, and therefore does not partake of society, is either a beast or a god*.”

–Aristotle

The long-asserted avowal of Aristotle is not an unfamiliar statement to society. As social beings, we are wired to connect (Lieberman, 2013), and our relationships are the essence of a happy and flourishing life (Valliant, 2002, 2012). Lieberman (2013) unmasked that our need to connect is as fundamental as our need for food, water, and shelter. Neuroscientists discovered that our brain responds to social pain and pleasure in the same powerful way as to physical pain and pleasure (Eisenberger, 2012; Hsu et al., 2015). The fact that we are wired as such means that our physical, emotional, and mental well-being depends on the positive interpersonal relationships in our everyday lives (Fishbane, 2007; Luong et al., 2011). In particular, our romantic relationships, which are seen as a near-universal need across cultures and various ages, have a powerful influence on our well-being (Jankowiak and Fisher, 1992; Kansky, 2018; Fletcher et al., 2019).

Evidence also recognizes that the impact of the relationship of an individual on well-being is contingent on the cultural values, orientation, and norms of a person germane to the social and interaction context in which the relationship exists (Kim et al., 2008). In general, positive psychology research studies around the world have been largely based on Caucasian samples, and more research is recommended to explore diversity in the science of positive psychology (Rao and Donaldson, 2015). Yet, the influence of culture on specific patterns of positive interactions regarding the realm of dating relationships in Malaysia and throughout Asia is relatively untapped. Notably, there has been a gap with limited positive psychology research and practice in Malaysia (Hashim, 2013; Hendriks et al., 2019).

The way an individual responds during couple conflicts (accommodation response) (i.e., Rusbult et al., 1991; Crowley, 2006) and toward disclosure of good news by a partner (capitalization response) (i.e., Gable et al., 2004, 2006) has been linked to relationship satisfaction and stability. More specifically, constructive accommodation and capitalization responses through couple conflicts and triumphs are associated with greater relationship well-being (Gable et al., 2004). Although the accommodation and capitalization communication processes are part and parcel of our everyday lives, these two processes have been examined separately and dominated by the Western perspectives in past research. Prior work has suggested that Western and Asian cultures differ in expressing and perceiving beneficial communication behaviors (e.g., Wang et al., 2010; Choi et al., 2019). Of concern, it is still unclear which accommodation and capitalization responses matter the most from an Asian lens thus far. To date, there is no research examining these interconnected variables simultaneously in Malaysia and across Asia. In this exploratory study, two forms of communication processes, accommodation, and capitalization were explored to disentangle the unique associations with relationship satisfaction, contributing to positive psychology insights for enriching relationships in an Asian context, specifically in Malaysia. Since Malaysia is a country with a melting pot of ethnicities and unique historical influences (Nagaraj et al., 2015; Park, 2015; The Malaysian Administrative Modernisation Management Planning Unit, 2016), this study may offer a different and novel positive psychology perspective to the constructs understudied. This study also sought to understand the moderating influence of culture in terms of self-construal on the link between these two communication processes and relationship satisfaction.

## Literature Review

### Romantic Relationships, Satisfaction, and Communication

There has been a great deal of literature examining overall relationship satisfaction and its consequences due to the considerable impact of romantic relationships on well-being (Karney and Bradbury, 1995; Bradbury et al., 2000; Dush and Amato, 2005; Fincham and Beach, 2010; Gomez-Lopez et al., 2019). When relationships are satisfying and fulfilling, couples are happier and healthier (Proulx et al., 2007), but when thwarted, other pillars of well-being can be jeopardized, such as mortality (Robles et al., 2014) and mental health, for example, increase in depression and anxiety symptoms (Snyder et al., 2005). Notably, the most prominent research on relationship satisfaction has been The Harvard University Adult Study of Development, the lengthiest longitudinal study in the positive psychology literature of the world with more than 80 years of research. This research called to the attention of audiences widespread regarding the importance of relationship satisfaction for flourishing lives as they found individuals in more satisfying marriages at age 50 had greater mental, emotional, and physical health at age 80 (Valliant, 2002, 2012; Waldinger and Schulz, 2010; Waldinger et al., 2014). Hence, not surprisingly, couple satisfaction has been viewed as the gold standard for assessing interventions to alleviate relationship distress and sustain thriving relationships (Fincham et al., 2018).

Relationship satisfaction has often been referred to as the global relationship measure (Tam et al., 2011a) and tends to be used interchangeably in the literature with terms such as relationship success, well-being, happiness, adjustment, and quality of a relationship (e.g., Vangelisti, 2004; Fincham and Rogge, 2010; Fincham et al., 2018). A satisfying relationship has been identified as a significant predictor of relationship well-being and longevity (Barnes et al., 2007; Ruffieux et al., 2014), yet it can feel like an unsolvable riddle to many couples. Given the strong predictive connection between relationship satisfaction and important life implications, it is critical to explore why some relationships lead to satisfaction and some fail? Why does a once loving and promising relationship break down over time? Interestingly, longitudinal (Karney and Bradbury, 1995; Gottman and Silver, 1999; Byers, 2005) and cross-sectional (Woodin, 2011) studies have unearthed that communication is pivotal in solving this riddle.

Communication has been found to be the bedrock or the “heart” in supporting and promoting relationship satisfaction (Gottman and Krokoff, 1989; Gottman and DeClaire, 2002; Markman et al., 2010; Hiew et al., 2016; Ogolsky et al., 2017), with recent findings linking satisfaction with constructive responses in conflicts (accommodation) and sharing of personal triumphs (capitalization). Within the Malaysian context, good communication has been reported as a core contributing factor to harnessing a happy and satisfying marriage (Abidin, 2019; Noor et al., 2019), thereby making a lack of effective communication and misunderstandings being one of the main reasons for relationship dissolutions (National Population Family Development Board Malaysia., 2016). On top of that, marital research experts have suggested that it is not the sheer frequency of positive to negative communication behaviors that influences the satisfaction of a couple, but the ratio of positive behaviors outweighing negative behaviors, 5:1, that leads to satisfaction (Gottman and Levenson, 1992; Gottman and Gottman, 2017). However, what is viewed as positive communication and rewarding in Western cultures may look different in Asian cultures.

Researchers over the years have argued that Western and Asian cultures differ in how they express and perceive beneficial communication behaviors (e.g., Wang et al., 2010; Williamson et al., 2012; Yum et al., 2015; Wang and Lau, 2018; Rajaei et al., 2019). Further, Finkle et al. (2017) have also surmised that favorable responses need to be tailored to the unique situational context of the couple and that responsiveness (i.e., showing understanding, care, and validation) would not be entirely universal to all circumstances. Thus, the maintenance of a satisfying relationship cannot be fully understood and appreciated without sufficient knowledge of the cultural underpinnings of communication in romantic relationships and specific situational contexts. While a few studies have examined certain variables and couple satisfaction in Malaysia (e.g., Hoesni et al., 2016; Abdullah et al., 2017; Abidin et al., 2018), knowledge about the psychology of positive communication processes through conflicts and triumphs and how culture impacts these interactional processes are rather oblique.

### Communication and Self-Construal

An underlying assumption of this current research was that individuals who vary in culture in terms of self-construal also differ in the way they perceive beneficial responses of their partner. Culture influences the behavior of an individual indirectly through molding personality dispositions such as self-construal (Yum, 2004), and research has shown that the variations in communicative behaviors could be explained by considering self-construal (Markus and Kitayama, 1991). Self-construal signifies the culturally contingent beliefs, feelings, and actions of an individual related to the understanding of the self as associated to others, in terms of members of in-groups (interdependence; InterSC) or separate from others (independence; IndSc) (Markus and Kitayama, 1991; Cross et al., 2011). The dominant self-construal of an individual is fundamentally driven by an individualism-collectivism cultural environment (Triandis, 1995). Generally, Western cultures adopt individualistic values while Eastern cultures are described to hold collectivistic values (Hofstede, 2001). In individualistic societies, people lean toward developing an independent self (IndSc) and value unique feelings and ideas, where asserting personal desires, goals, and emotions are favorable (Markus and Kitayama, 1991). Contrarily, people in collectivistic societies tend to view the self as interdependent with values of relational harmony and are socialized to accommodate groups and subordination of personal desires (Morling et al., 2002).

While verbal expression and direct communication is common practice in IndSc dominant cultures (Kim and Markus, 2002), indirect and less expressive communication is preferred by InterSc cultures as verbalizing internal states may be seen as disruptive to group harmony (Kim and Sherman, 2007; Ma-Kellams and Blascovich, 2012). Therefore, people in Malaysia who are traditionally in a collectivist society (Hofstede, 2001; Ting and Ying, 2013) would presumably hold a more dominant InterSC and may use and prefer different communicative behaviors compared to individualistic societies. Evidently, Yum et al. (2015) found that Malaysians use less direct communication and self-disclosure to express their commitment and affection compared to Americans. Moreover, Malaysians place great weight on the collective well-being (Kennedy, 2002) and tend to practice caution and indirectness in daily communication (Bakar et al., 2007, 2014). Thus, the Malaysian culture inhibits assertiveness and confrontational behaviors to maintain harmony within relationships (Kennedy, 2002). This knowledge suggests that the culture of an individual with respect to the dominant self-construal may impact the type of approach and how efficacious communication behaviors are anticipated to be. Thus, the positive association between active communication behaviors (e.g., positive verbal expression) and the negative association between passive communication behaviors (e.g., withdrawal from the conversation), with relationship satisfaction in Western societies, may not be universal to Asian societies, particularly in the Malaysian cultural atmosphere.

### Communication Through Conflicts: Accommodation, Self-Construal, and Satisfaction

Unraveling the mystery surrounding the riddle of achieving satisfying relationships is even more perplexing when communicative behaviors may operate differently in a different context. A growing body of literature has examined communicative processes within conflictual contexts, namely, accommodative behaviors, which is described as inhibiting natural reflexes of reacting negatively to the transgressions of a partner and instead respond positively (Rusbult et al., 1991; Overall and Sibley, 2008; Overall et al., 2010). Irrespective of how compatible partners are in a relationship, conflict is inevitable (Rusbult et al., 1991), and all partners will occasionally behave in an unpleasant manner such as yelling or saying hurtful remarks (Yovetich and Rusbult, 1994; Kilpatrick et al., 2002; Crowley, 2006). To protect the quality of the romantic relationship in the long run, couples must override the urge to act destructively during conflicts, hence the term accommodation (Campbell and Staton, 2013). The accommodation model is measured along two dimensions and is differentiated into four types of responses: active-constructive (discussing problems and attempts to resolve the problem), passive-constructive (silently forgives and waits for things to improve), active-destructive (criticizing and threatening to leave the partner), and passive-destructive (ignoring the partner and problem) (Rusbult et al., 1982, 1991; Overall et al., 2010).

Consider this example scenario in daily life where Liam raises his voice toward Camelia in a conversation after a long day at work. Camelia “bites the bullet” and reacts active-constructively or passive-constructively by either asking him whether he needs to talk about his day or calmly shrugging it off. Constructive responses, like that of Camelia, have been linked to better couple functioning in prior work (Rusbult et al., 1982, 1991). To elaborate more, the pioneer study by Rusbult et al. (1991) revealed that responding in both an active or passive constructive manner during conflicts and toward the transgressions of a partner preserves relationship satisfaction and stability. However, a later study discovered that only active-constructive responses were associated to elevated feelings of closeness, value, relationship stability, and satisfaction (Overall et al., 2010). Conversely, passive-constructive responses did not produce the same benefits, were less noticed, and results were parallel to harmful implications of destructive responses (Overall et al., 2010). Such counterintuitive findings carry doubts and uncertainty surrounding the passive-constructive communication in conflicts within Western society. There is even greater ambiguity regarding these communicative behaviors in Asian society, whereupon the accommodation research is rather scarce.

When discussing problems, those with InterSc (i.e., Asian societies) have been typically associated with an indirect communication style (Gudykunst and Matsumoto, 1996), where the listener is expected to deduce the unexpressed meaning of the speaker through non-verbal cues (Ting-Toomey, 1999). In contrast, those with IndSc (i.e., Western societies) tend to disclose thoughts and feelings more explicitly (Ting-Toomey, 1999). Other empirical support exists in the view that members from Asian cultures deter from expressing distress as it may threaten relationship ties due to the possibility of burdening others or conflicts (Wang et al., 2010), which prompts them to oblige to others more (Oetzel and Ting-Toomey, 2003). Of the few studies regarding accommodation in Asia, the only exception in the literature examining self-construal in accommodative dilemmas is by Yum (2004) on 397 individuals from the United States, Hawaii, and Korea. Yum (2004) found that both IndSC and InterSC were inclined to respond in an active-constructive and passive-constructive manner in dating relationships, suggesting that accommodation may be a culturally universal behavior in dating relationships. However, those with InterSC enacted more passive-constructive communicative behaviors. Interestingly, Yum (2004) also found that some participants were bicultural (high in both InterSC and IndSC) and marginal (low in both InterSC and IndSC), with biculturals being better communicators compared to those with high InterSC. Yum (2004) explained that the disparities and new findings may be due to modernization, implying that behaviors of people are influenced by the degree of democratization, industrialization, and westernization within the environmental culture they live in. While a few studies in Malaysia have investigated couple communication tactics in conflicts (e.g., Tam et al., 2011b; Abdullah et al., 2017), there is no literature focused directly on accommodation processes. It is also not yet known to what extent dating partners in Malaysia may perceive beneficial responses in managing conflicts. Henceforth, based on the findings and reasoning above, Malaysians may find both active and passive constructive accommodation responses as favorable reactions during conflicts, which would positively relate to their relationship satisfaction.

### Communication Through the Good Times: Capitalization, Self-Construal, and Relationship Satisfaction

Similar desirable relationship outcomes exist for positive relational communication. A wealth of research has focused on negative relational processes such as conflict, problem solving, and criticism (e.g., Rusbult et al., 1991; Gottman, 1998; Johnson et al., 2005), while the positive relational processes have often been left to lie fallow. Recent work has finally stressed the advantages of the positive side of relationships (Gable et al., 2004, 2006; Lambert et al., 2012; Pagani et al., 2020), providing a fresh positive psychology perspective of not only minimizing threat or lasting harm to satisfaction (i.e., conflict management) but also integrating relationship enhancement processes (i.e., positive communication, and responsiveness) (Ogolsky et al., 2017; Warren et al., 2017). In particular, the process of capitalization, which is a practice of communicating personal positive events to others (Langston, 1994; Gable et al., 2004), has begun to gain much attention and is a focus of this current study. This gap is noteworthy as past studies have established that individuals share more positive events with others daily, with an estimation of 60 to 80% more compared to negative events. Hence, showing that capitalization opportunities and positive events occur more often than negative events and conflicts in everyday life (Gable et al., 2004; Gable and Haidt, 2005; Gable and Reis, 2010). In fact, responses toward positive events were a better predictor for relationship well-being than responses toward negative events (Gable et al., 2006).

Happy events (e.g., a promotion, a great cup of coffee, and compliments from others) usually motivates social retelling of those positive circumstances (Peters et al., 2018). For example, when Camelia receives a promotion at work, she would be motivated to share this news with her partner Liam. Provided that the reaction of Liam to the good news of Camelia was responsive, the capitalization process can be contagious, benefiting both parties and would promote future capitalization attempts, positive responses, and lasting relationship well-being (Peters et al., 2018). Among the various ways of responding to positive events, Gable et al. (2004) adapted the accommodation model to four types of capitalization responses. Hence, constructive capitalization responses can either be active (showing interest and enthusiasm) or passive (understating the event), whereas destructive capitalization responses may be either active (criticizing and invalidating event) or passive (showing disinterest and ignoring the event) (Gable et al., 2004, 2006). To provide more context, the first investigation of capitalization in dating couples found that only an active-constructive response of a partner had a positive correlation with relationship satisfaction while active-destructive, passive-destructive, and passive-constructive responses showed an opposite effect for relationship satisfaction (Gable et al., 2004). Other studies have also found an association between relationship satisfaction and enthusiastic responses to the triumphs of a partner (e.g., Logan and Cobb, 2013, 2016; Woods et al., 2015), but are all skewed to the Western perspectives.

Of note, a favorable response in one culture may not look the same in another as cultural differences could facilitate or impede capitalization processes (Choi et al., 2019). Wang et al. (2010) documented that Asian-Americans utilized support and perceived support for positive events as less helpful than their European-American counterparts. This result may be due to East Asian cultures viewing humility as prosocial, whereas capitalization can be seen as an individual “showing off,” being boastful, and threatening harmony (Markus and Kitayama, 1991; Yamagishi, 2011; Choi et al., 2019). Nevertheless, considering that being understood and validated by other people is considered the quintessence of the interdependent self (Markus and Kitayama, 1994), supportive and constructive responses may still carry weight for East Asians (Choi et al., 2019). A less emotionally expressive response, such as a passive-constructive response, could allow people from a collective culture to capitalize and experience appropriate emotional support without being overzealous, which might make them feel uncomfortable (Taylor et al., 2007). Accordingly, research by Kim (2015) on cultural distinctions in capitalization responses between Asian-Americans (AAs) and European-Americans (EAs) revealed that there were no differences in InterSC and only marginal significant differences in IndSC between cultural groups. For a satisfying relationship, both cultural groups favored active-constructive responses the most, implying the global advantages of active-constructive responses. Moreover, AAs did not respond adversely to passive-constructive responses, presumably indicating that a passive-constructive response may not be a detrimental response for AAs. Currently, it is unclear if replicable findings will be seen for individuals who are in dating relationships in Malaysia. To our knowledge, there is no research identified examining capitalization and romantic relationships in Asia, but there was one research that utilized a Chinese translated capitalization scale within father-child relationships of college students in China (Guo et al., 2018). Guo et al. (2018) reported both active and passive responses of fathers were positively linked to the intrapersonal health and well-being of their children, whereas the reverse impact was found for the two destructive responses. Thus, based on prior empirical and theoretical work, it is fair to say that there could be possible differences between perceived favorable responses for capitalization processes in romantic relationships between the Western and Asian cultures.

### Current Study

Taken together, these lines of studies suggest that relationship satisfaction is not governed by the simple occurrence of conflict or positive events in the lives of a couple but by the capability of the couple to communicate about those events effectively. As evident from prior literature, the communication processes of both accommodation and capitalization responses may vary across cultures. Each form of communication process provides an important piece to move closer to understanding the relationship satisfaction enigma. However, both accommodation and capitalization have been researched independently and dominated in Western countries, which creates a drawback in comprehending the full picture of relationship functioning in Asian countries. Correspondingly, self-construal is also seen to be a moderating influence on both accommodation and capitalization processes. To the best knowledge of the researchers, to date, there is no identified research investigating these variables simultaneously, and the only study found in peer-reviewed publications was by Gable et al. (2004). On the one hand, the researchers discovered that only responding in an active-constructive manner toward the capitalization attempts of a partner correlated positively with satisfaction, while the other three responses yielded opposite effects. On the other hand, Gable et al. (2004) uncovered that both active and passive constructive accommodation responses during conflicts were positively linked to satisfaction with good agreement to the initial findings of Rusbult et al. (1991). Thus, results suggest that these communicative behaviors may not be parallel to each other and may depend on the situational context.

However, Gable et al. (2004) did not examine any cultural aspects, and their results were weighted toward the Western perspective. Thus, this current study would be the first not merely in Malaysia but the overall literature to further deepen the results of Gable et al. (2004) on “good relationship behavior.” Herewith, this present study conducted both correlations and hierarchical analyses. At first, accommodation and capitalization responses were analyzed separately to examine how each response uniquely predicted satisfaction. Subsequently, both accommodation and capitalization were examined simultaneously in the regression model to ascertain which responses are better predictors of satisfaction. Using these methods would allow a better understanding of the unique effects of each accommodation and capitalization response on relationship satisfaction with the complements of culture. It is also the hope of this exploratory study to shed light on how the distinct culture of Malaysian young dating individuals influences their communication behaviors to augment thriving relationships, withal providing an Asian comparative data. Furthermore, most studies have researched on the self-evaluation of an individual to their own communication; though often, one is a very poor judge of one's own communicative behavior (Rusbult et al., 1991). Therefore, this present study would investigate how one perceives the accommodation and capitalization responses of their partner and how these perceptions relate to their overall relationship satisfaction.

With the limitations and dearth of literature, the present study aimed to bridge the gaps by exploring the relationship between the two communication processes, (1) accommodation and (2) capitalization, on relationship satisfaction in Malaysian young adults. This study also aimed to examine whether interdependent self-construal moderates and explains the differences in perceived communication behaviors, which in turn effects the level of relationship satisfaction. In broader terms, this research anticipated that active and passive constructive responses would positively predict satisfaction, whereas active and passive destructive responses would negatively predict satisfaction for both accommodation and capitalization processes. With greater depth, the following research questions and hypotheses are postulated:

1. **Research Question 1**
2. Do the eight types of perceived communication behaviors, in terms of accommodation (4 types) and capitalization (4 types) responses predict relationship satisfaction?
  *Hypothesis 1a (H1a)*: Perceived active-constructive accommodation responses will positively predict relationship satisfaction.
  *Hypothesis 1b (H1b)*: Perceived passive-constructive accommodation responses will positively predict relationship satisfaction.
  *Hypothesis 1c (H1c)*: Perceived active-destructive accommodation responses will negatively predict relationship satisfaction.
  *Hypothesis 1d (H1d)*: Perceived passive-destructive accommodation responses will negatively predict relationship satisfaction.
  *Hypothesis 1e (H1e)*: Perceived active-constructive capitalization responses will positively predict relationship satisfaction.
  *Hypothesis 1f (H1f)*: Perceived passive-constructive capitalization responses will positively predict relationship satisfaction.
  *Hypothesis 1g (H1g)*: Perceived active-destructive capitalization responses will negatively predict relationship satisfaction.
  *Hypothesis 1h (H1h)*: Perceived passive-destructive capitalization responses will negatively predict relationship satisfaction.
3. **Research Question 2**
4. To what extend does interdependent self-construal moderate the relationship between perceived accommodation and capitalization communication behaviors on relationship satisfaction?
  *Hypothesis 2a (H2a)*: As an exploratory hypothesis, interdependent self-construal will moderate the relationship between the four perceived accommodation responses and relationship satisfaction.
  *Hypothesis 2b (H2b)*: As an exploratory hypothesis, interdependent self-construal will moderate the relationship between the four perceived capitalization responses and relationship satisfaction.

## Methods

### Participants

Participants were 139 individuals (46 males, 93 females) recruited on a voluntary basis with online advertisements. A priori power analysis and F-test, linear multiple regression with a fixed model, and *R*^2^ deviation from zero was calculated through the G^*^ Power 3.1 software. The results demonstrated that the sample size was sufficient to detect a significant effect size with 80% power (*f*^2^ = 0.15, α = 0.05, two-tailed) (Faul et al., 2007). Eligibility criteria included the criteria for participants to be Malaysian, above 18 years old, fluent in English and in a romantic relationship for a minimum of 3 months. Initially, there were 179 participants, however, 40 participants were excluded as they did not meet the required criteria for the survey (e.g., minimum 3 months of relationship length) and possibly due to the length of the survey and lack of compensation and token of appreciation. Participants ranged from 18 to 30 years of age (*M* = 23.15, *SD* = 2.42) and age of partners ranged from 18 to 35 years old (*M* = 23.93, *SD* = 3.39). The average romantic relationship length of participants was 2.73 years (*SD* = 2.34). Other demographic information of participants is summarized in Table 1.

**Table 1:** Participant demographics (*N* = 139).

	***N***	**%**
**Ethnicity**		
Malay	28	20.1
Chinese	51	36.7
Indian	37	26.6
Others	23	16.6
**Relationship status**		
Casual dating	20	14.4
Seriously dating	98	70.5
Engaged	17	12.2
Others	4	2.9
**Sexual orientation**		
Heterosexual	132	95
Bisexual	2	1.4
Others	2	1.4
Prefer not to state	3	2.2
**Religion**		
Muslim	28	20.1
Christian	42	30.2
Buddhist	18	12.9
Hindu	20	14.4
Sikh	1	0.7
Atheist	6	4.3
Do not identify with a religion	15	10.8
Others	5	3.6
Prefer not to state	4	2.9
**Perceived economic status**		
Low	3	2.2
Low-middle	8	5.8
Middle	89	64.0
Middle-high	36	25.9
High	3	2.2
**Highest education level**		
Secondary school	8	5.8
Pre-university	19	13.7
Some undergraduate studies	22	15.8
Undergraduate	76	54.7
Postgraduate	14	10.0
**Student status**		
Studying full-time	49	35.3
Studying part-time	7	5.0
Not studying	83	59.7
**Employment status**		
Working full-time	75	53.9
Working part-time	24	17.3
Not working	40	28.8

### Procedure

Subsequent to approval of the study from the Human Research Ethics Committee of the university (MUHREC, Project Number 10606), the study was advertised online through voluntary and snowballing, non-probability sampling methods from December 2017 to January 2018. Numerous organizations and online mediums such as forums, discussion groups, non-governmental organizations, and health and education professionals were approached to advertise the research widely and recruit participants to promote sample diversity. Efforts were also made to foster inclusivity in several manners, for instance, by recruiting participants of different genders, relationship lengths, sexualities, and ethnicities. Additionally, participants were recruited on a voluntary basis instead of providing compensation for taking part in the study to minimize biases.

Participants were given an explanatory statement comprising the aim of the research, confidentiality and the anonymity of information collected. The explanatory statement also stated the rights of participants to withdraw from the study at any time before submitting their responses anonymously. Participants who voluntarily agreed to participate with consent implied, completed the research survey online through the Qualtrics site of the university. The research survey encompassed demographic background, relationship satisfaction, capitalization, accommodation, and self-construal questions. The duration time to complete the survey was ~30 min. Data of each participant was then merged into one data file and analyzed using IBM SPSS Statistics 25.0 software.

### Design

Exploratory cross-sectional research was conducted as an observation of variables at a single point of time. There were:

1. Two kinds of communication processes; accommodation and capitalization responses with the same four categories each as below, totaling up to eight predictors:
  - Active-constructive accommodation
  - Passive-constructive accommodation
  - Active-destructive accommodation
  - Passive-destructive accommodation
  - Active-constructive capitalization
  - Passive-constructive capitalization
  - Active-destructive capitalization
  - Passive-destructive capitalization
2. One moderator of self-construal:
  - Interdependent self-construal
3. One outcome:
  - Relationship satisfaction

### Assessment Tools

#### Relationship Satisfaction Measure

The Couples Satisfaction Index (CSI-16) has 16 items and measures the level of relationship satisfaction of an individual (Funk and Rogge, 2007). The CSI-16 has a variety of questions, all with a 5-point Likert-type scale (Funk and Rogge, 2007). The scores are calculated by tallying up the total points of the items, which can range between 0 and 81 (Funk and Rogge, 2007). Scores below 51.5 suggest distress in the relationship (Funk and Rogge, 2007). The CSI-16 showed high reliability with a Cronbach's alpha of 0.94 for both genders in European, Asian, and American cultures (Graham et al., 2011; Lee, 2013). It also showed strong convergent, construct validity, and greater power in recognizing different levels of satisfaction compared to other measures (Funk and Rogge, 2007). The Cronbach alpha for the overall scale in this present study was 0.95.

#### Capitalization Measure

The perceived responses to capitalization attempts (PRCA) scale consists of 12-items measuring the perceptions of the responses of a partner when shared with a positive event (Gable et al., 2004). The scale consists of three questions of each response type and are computed by tallying up each subscale, namely, active-constructive (3 items; e.g., “*My partner usually reacts to my good fortune enthusiastically*.”), passive-constructive (3 items; e.g., “*My partner tries not to make a big deal out of it, but is happy for me*.”), active-destructive (3 items; e.g., “*My partner often finds a problem with it*.”), and passive-destructive (3 items; e.g., “*My partner doesn't pay much attention to me*.”) responses (Gable et al., 2006). Participants rate each item using the line, “When I tell my partner about something good that has happened to me...”, using a 7-point scale from 1 (*not at all true*) to 7 (*very true*). PRCA demonstrated good reliability with men (α = 0.84) and women (α = 0.81) (Gable et al., 2006). Reliability analyses for this present study revealed an acceptable coefficient for the items devised to measure active-constructive (α = 0.61), passive-constructive (α = 0.65), active-destructive (α = 0.72), and passive destructive (α = 0.82), respectively.

#### Accommodation Measure

The accommodation scale is a 16-item measure that evaluates four perceived responses of a partner for each category: active-constructive (e.g., “*When I am rude to my partner, he/she tries to resolve the situation and improve conditions*.”), passive-constructive (e.g., “*When I do something thoughtless, my partner patiently waits for things to improve*.”), active-destructive (e.g., “*When I say something really mean, my partner threatens to leave me*.”), and passive-destructive (e.g., “*When I do something thoughtless, my partner avoids dealing with the situation*.”) to an individual's own adverse behavior (Rusbult et al., 1991). Items are scored on a 9-point Likert scale from 1 (*never does this*) to 9 (*constantly does this*). Total scores are calculated by totaling the four items of each subscale to gain the totals of each active-constructive, passive-constructive, active-destructive, and passive destructive responses (Gable et al., 2004). High reliability was shown with Cronbach's alpha (α = 0.83) (Crowley, 2006). Reliability for each item for this present study was also acceptable with active-constructive (α = 0.88), passive-constructive (α = 0.78), active-destructive (α = 0.73) and passive-destructive (α = 0.61), respectively.

#### Self-Construal Measure

The Singelis Self-Construal Scale (SCS) assesses the interdependent and independent self-construal of an individual (Singelis, 1994). The 30-item SCS has 15 interdependent and 15 independent items each. These 30 items are measured on a 7-point Likert scale from 1 (*strongly disagree*) to 7 (*strongly agree*). Responses on each subscale are averaged to obtain interdependent and independent scores separately with greater scores signifying greater self-construal (Hardin et al., 2004). A range of 0.60–0.73 Cronbach alpha reliabilities were found in Malaysia (Miramontes, 2011). Reliability coefficients in this present study were high for interdependent and independent self-construal with 0.83 and 0.76, respectively.

### Data Analysis

All data collected were analyzed descriptively through SPSS. Data cleaning was performed to check for outliers and missing values. Hierarchical multiple regression and moderation were conducted following discussion with statistical consultants knowledgeable about cross-cultural social psychology research. The assumptions for hierarchical multiple regression and moderation were assessed. After preliminary correlations were conducted, hierarchical multiple regressions were run to examine how capitalization and accommodation responses explain the variances in the relationship satisfaction score. Lastly, moderation analyses were run to assess whether interdependent self-construal moderates the relationship between the capitalization and accommodation responses and relationship satisfaction.

Given the theoretical and exploratory nature of this research, formal sensitivity analyses were not conducted as there was limited comparative data and models to compare the results. In preliminary analyses, the researchers have explored the results by entering only significant accommodation and capitalization responses from the correlations to the hierarchical regression and found little change in the *R*^2^ compared to the current findings with all eight responses in the model. These results may be due to the additional responses not indicating a substantial predictive value, in which the rationales have been explained in the discussion section. Additionally, the researchers have inspected the results by changing the order of the input stages for the hierarchical regression, and similar results were demonstrated.

## Results

Assumption tests were run prior to inferential analysis. Missing values analysis found missing data and these participants were omitted from further analysis. A 22.3% rate of non-participation was discovered due to participants not meeting the requirements of the research survey. Two univariate outliers were identified for passive-destructive capitalization based on the criteria of z-score ± 3.29 and were winsorized (Field, 2013). Normality analysis revealed that multiple variables violated normality; however, based on the central limit theorem, the sample size was deemed adequately large to assume normality (Field, 2013). Assumptions of multicollinearity and singularity were assumed to be met, established upon the criteria of Tolerance not lower than 2 and VIF not >10 (Field, 2013). Lastly, visual inspections of residual scatter plots showed that data were both linearly distributed and homoscedastic.

Table 2 presents the descriptive statistics of the full sample (*N* = 139). Despite targeted efforts to include both genders, it is also important to note that this sample had a higher percentage of female to male participants (66.9–33.1%). Additionally, the cut-off score for relationship satisfaction is 51.5 whereby anything below this score suggests notable relationship dissatisfaction (Funk and Rogge, 2007). 73.4% of participants were above this cut-off score signifying that most participants in this sample are in relatively satisfied relationships.

**Table 2:** Descriptive statistics of all main variables.

	***N***	***M***	***SD***
Relationship satisfaction	139	57.04	12.88
Active-constructive capitalization	139	15.67	3.92
Passive-constructive capitalization	139	11.15	4.69
Active-destructive capitalization	139	8.68	4.41
Passive-destructive capitalization	139	6.13	3.97
Active-constructive accommodation	131	20.50	8.05
Passive-constructive accommodation	131	18.40	7.61
Active-destructive accommodation	131	6.80	6.56
Passive-destructive accommodation	131	8.38	6.30
Interdependent self-construal	123	4.99	0.76
Independent self-construal	123	5.15	0.69

### The Relationship Between Perceived Accommodation and Capitalization Responses on Relationship Satisfaction

Bivariate correlations were conducted for the accommodation and capitalization variables with relationship satisfaction, shown in Table 3. The examination of correlation between perceived accommodative responses and relationship satisfaction showed that there was a positive correlation between Active-constructive accommodation responses and relationship satisfaction (*r* = 0.39, *p* < 0.001). The Passive-constructive accommodation responses had a *r* = 0.14, *p* = 0.056. Both Active-destructive (*r* = −0.36, *p* < 0.001) and Passive-destructive (*r* = −0.40, *p* < 0.001) accommodation responses had a negative correlation with relationship satisfaction. Thus, in this preliminary analysis, out of the four accommodation responses, only three responses (active-constructive, active-destructive, and passive-destructive) appeared to have a significant association with satisfaction.

**Table 3:** Correlations for accommodation and capitalization responses with relationship satisfaction.

**Responses**	**Relationship satisfaction ***r*****
Active-constructive accommodation	0.39^**^
Passive-constructive accommodation	0.14
Active-destructive accommodation	−0.36^**^
Passive-destructive accommodation	−0.40^**^
Active-constructive capitalization	0.44^**^
Passive-constructive capitalization	0.02
Active-destructive capitalization	−0.13
Passive-destructive capitalization	−0.39^**^

** *p < 0.001*.

Correlations for perceived capitalization responses and relationship satisfaction revealed that only two responses, Active-constructive and Passive-destructive capitalization responses indicated a significant link to satisfaction. Active-constructive responses (*r* = 0.44, *p* < 0.001) and Passive-constructive responses (*r* = 0.02, *p* = 0.429) had a positive correlation with relationship satisfaction. Both passive-destructive responses (*r* = −0.39, *p* < 0.001) and active-destructive responses (*r* = −0.13, *p* = 0.060) had an inverse association with relationship satisfaction. Moreover, correlations between accommodation and capitalization were assessed and revealed the strength of association ranging from 0.01 to 0.52.

Addressing research question and hypotheses 1, hierarchical multiple regression analyses were performed to further investigate the directionality of the eight accommodation and capitalization communicative responses and determine which of those communication variables uniquely contributed to the prediction of relationship satisfaction. At first, accommodation and capitalization responses were analyzed separately to examine how each response uniquely predicted satisfaction. Subsequently, both accommodation and capitalization were examined simultaneously in the regression model to ascertain which responses were better predictors of satisfaction. In order to accurately examine their unique influence of relationship satisfaction, demographic variables of age, gender, and relationship length which may have effects on relationship satisfaction were controlled by entering them first in stage one of all the hierarchical multiple regression analyses. Gender was held constant because the ratio of female to male participants was larger.

The first hierarchical regression executed was on the four accommodation responses as predictors and relationship satisfaction as the outcome variable (see Table 4). As aforementioned, in stage 1, gender, age, and relationship length were entered and did not contribute significantly to relationship satisfaction, *F*_(3,127)_ = 1.67, *p* = 0.176, only accounting for a difference of 0–3.8% in the variability of relationship satisfaction. Next, Active-constructive accommodation, Passive-constructive accommodation, Active-destructive accommodation, and Passive-destructive accommodation were entered in stage 2, which suggested an increase in predictive capacity of relationship satisfaction by 26.3%, *F*_(4,123)_ = 7.56, *p* < 0.001. A large effect size, Cohen's *f*^2^ = 0.38 was demonstrated between the set of predictors in stage 1 and stage 2 (Cohen, 1988). Among the four accommodation responses, only two responses, Active-constructive and Passive-destructive accommodation, emerged as unique predictors of satisfaction, with Active-constructive accommodation [β = 0.34, 95% CI (0.19, 0.87), *p* < 0.05] recording a stronger relationship with satisfaction compared to Passive-destructive accommodation [β = −0.24, 95% CI (−0.87, −0.09), *p* < 0.05]. In contrast, Passive-constructive accommodation [β = −0.05, 95% CI (−0.42, 0.25), *p* = 0.617] and Active-destructive accommodation [β = −0.15, 95% CI (−0.65, 0.07), *p* = 0.118] revealed to have less predictive value toward relationship satisfaction.

**Table 4:** Accommodation responses as predictors of relationship satisfaction.

	**Predictor variable**	***B***	**95% CI**	***SE B***	**β**	***t*** **-value**	***p***	***R*** ^**2**^	***R***^**2**^ **change**	***p***
Model 1								0.03	0.03	0.176
	Gender	−0.54	(−5.15, 4.07)	2.3	−0.02	−0.23	0.818			
	Age	−0.74	(−1.65, 0.172)	0.46	−0.14	−1.60	0.111			
	Relationship length	0.86	(−0.10, 1.82)	0.49	0.15	1.76	0.080			
Model 2								0.30	0.26	0.000^**^
	Gender	−5.6	(−9.88, −1.36)	2.1	−0.21	−2.6	0.010			
	Age	−0.37	(−1.18, 0.42)	0.40	−0.07	−0.94	0.352			
	Relationship length	0.71	(−0.14, 1.56)	0.43	0.13	1.66	0.100			
	Active-constructive accommodation	0.53	(0.19, 0.87)	0.17	0.34	3.10	0.002^*^			
	Passive-constructive accommodation	−0.09	(−0.42, 0.25)	0.17	−0.05	−0.50	0.617			
	Active-destructive accommodation	−0.29	(−0.65, 0.07)	0.18	−0.15	−1.57	0.118			
	Passive-destructive accommodation	−0.48	(−0.87, −0.09)	0.20	−0.24	−2.45	0.016^*^			

* *N = 131, p < 0.05*,

** *p < 0.001*.

The second hierarchical regression model explored the unique association of the four capitalization responses on relationship satisfaction (see Table 5). Similarly, age, gender, and relationship satisfaction were entered in stage 1 [*R*^2^ = 0.03, *F*_(3,135)_ = 1.44, *p* = 0.234]. Introducing the Active-constructive capitalization, Passive-constructive capitalization, Active-destructive capitalization, and Passive-destructive capitalization responses to stage 2 explained an additional 24.3% of variability of relationship satisfaction, significantly increasing the predictive capacity of relationship satisfaction, *F*_(4, 131)_ = 7.05, *p* < 0.001. A Cohen *f*^2^ = 0.33 value was found, signifying a moderate effect size between the set of predictors in stage 1 and stage 2 (Cohen, 1988). Both constructive responses showed contributing value in predicting relationship satisfaction, with Active-constructive capitalization [β = 0.33, 95% CI (0.52, 1.65), *p* < 0.001] demonstrating a stronger relationship than Passive-constructive capitalization [β = 0.17, 95% CI (0.00, 0.92), *p* < 0.05]. For the two destructive responses, Active-destructive capitalization [β = −0.04, 95% CI (−0.66, 0.41), *p* = 0.648] displayed low predictive capacity for satisfaction, while Passive-destructive capitalization [β = −0.25, 95% CI (−1.42, −0.16), *p* < 0.05] indicated strong predictive value toward relationship satisfaction.

**Table 5:** Capitalization responses as predictors of relationship satisfaction.

	**Predictor variable**	***B***	**95% CI**	***SE B***	**β**	***t*** **-value**	***p***	***R*** ^**2**^	***R***^**2**^ **change**	***p***
Model 1								0.03	0.03	0.234
	Gender	−0.71	(−5.28, 3.87)	2.31	−0.03	−0.31	0.760			
	Age	−0.52	(−1.42, 0.38)	0.45	−0.10	−1.15	0.254			
	Relationship length	0.87	(−0.06, 1.81)	0.47	0.16	1.85	0.066			
Model 2								0.27	0.24	0.000^**^
	Gender	−1.1	(−5.18, 2.98)	2.06	−0.04	−0.53	0.594			
	Age	−0.35	(−1.16, 0.45)	0.41	−0.07	−0.87	0.388			
	Relationship length	0.64	(−0.20, 1.48)	0.43	0.12	1.5	0.136			
	Active-constructive capitalization	1.09	(0.52, 1.65)	0.29	0.33	3.8	0.000^**^			
	Passive-constructive capitalization	0.46	(0.00, 0.92)	0.23	0.17	2.0	0.048^*^			
	Active-destructive capitalization	−0.12	(−0.66, 0.41)	0.27	−0.04	−0.46	0.648			
	Passive-destructive capitalization	−0.79	(−1.42, −0.16)	0.32	−0.25	−2.48	0.015^*^			

* *N = 139, p < 0.05*,

** *p < 0.001*.

Independently, accommodation and capitalization demonstrated to contribute to relationship satisfaction. However, the next part of the analysis was to explore all eight accommodation and capitalization responses simultaneously. Analogous to the two previous regression models, gender, age, and relationship length were entered in stage 1 and appeared non-significant with *R*^2^ = 0.3, *F*_(3,127)_ = 1.67, *p* = 0.176. Introducing the four accommodation responses to stage 2 explained an additional 26.3% of variation in satisfaction, *F*_(4,123)_ = 7.55, *p* < 0.001, and a large effect size of Cohen *f*^2^ = 0.38 was found between stage 1 and stage 2 (Cohen, 1988). Finally, adding the four capitalization responses to the regression model explained an additional 5.6% of variation in satisfaction and this change in *R*^2^ was also significant with *F*_(4,119)_ = 6.01, *p* < 0.001. The effect size between the set of predictors of stage 2 and stage 3 was small, Cohen *f*^2^ = 0.09 (Cohen, 1988). It can be seen in Table 6 that when all eight accommodation and capitalization responses were measured simultaneously as predictors of relationship satisfaction in stage 3, merely an active-constructive capitalization response was found as a strong incremental predictor to relationship satisfaction with β = 0.25, 95% CI (0.18, 1.4), *p* < 0.05. A marginal positive predictive value was found for the active-constructive accommodation β = 0.22, 95% CI (−0.00, 0.71), *p* = 0.054. The rest of the accommodation and capitalization responses were found to provide minimal contribution, suggesting less predictive value for satisfaction.

**Table 6:** Accommodation and capitalization responses as predictors of relationship satisfaction.

	**Predictor variable**	***B***	**95% CI**	***SE B***	**β**	***t*** **-value**	***p***	***R*** ^**2**^	***R***^**2**^ **change**	***p***
Model 1								0.03	0.03	0.176
	Gender	−0.54	(−5.15, 4.07)	2.3	−0.02	−0.23	0.818			
	Age	−0.74	(−1.65, 0.172)	0.46	−0.14	−1.60	0.111			
	Relationship length	0.86	(−0.10, 1.82)	0.49	0.15	1.76	0.080			
Model 2								0.30	0.26	0.000^**^
	Gender	−5.6	(−9.88, −1.36)	2.1	−0.21	−2.6	0.010			
	Age	−0.38	(−1.18, 0.42)	0.40	−0.07	−0.94	0.352			
	Relationship length	0.71	(−0.14, 1.56)	0.43	0.13	1.66	0.100			
	Active-constructive accommodation	0.53	(0.19, 0.87)	0.17	0.34	3.10	0.002^*^			
	Passive-constructive accommodation	−0.09	(−0.42, 0.25)	0.17	−0.05	−0.50	0.617			
	Active-destructive accommodation	−0.29	(−0.65, 0.07)	0.18	−0.15	−1.57	0.118			
	Passive-destructive accommodation	−0.48	(−0.87, −0.09)	0.20	−0.24	−2.45	0.016^*^			
Model 3								0.36	0.05	0.000^**^
	Gender	−03.61	(−0.79, 0.73)	2.2	−0.14	−1.65	0.102			
	Age	−0.37	(−1.16, 0.42)	0.40	−0.07	−0.93	0.352			
	Relationship length	0.67	(−0.17, 1.51)	0.43	0.12	1.59	0.116			
	Active-constructive accommodation	0.35	(−0.01, 0.71)	0.18	0.22	1.95	0.054			
	Passive-constructive accommodation	−0.18	(−0.51, 0.16)	0.17	−0.11	−1.06	0.291			
	Active-destructive accommodation	−0.24	(−0.62, 0.15)	0.19	0.12	−1.23	0.223			
	Passive-destructive accommodation	−0.35	(−0.76, 0.07)	0.21	−0.17	−0.1.66	0.099			
	Active-constructive capitalization	0.79	(0.18, 1.4)	0.31	0.25	2.55	0.012^*^			
	Passive-constructive capitalization	0.44	(−0.03, 0.90)	0.24	0.16	1.86	0.065			
	Active-destructive capitalization	−0.09	(−0.64, 0.46)	0.28	−0.03	−0.33	0.743			
	Passive-destructive capitalization	−0.25	(−0.92, 0.41)	0.34	−0.08	−0.75	0.453			

* *N = 131, p < 0.05*,

** *p < 0.001*.

The communication responses were entered in this order given that accommodation is a more well-known process as a contributor to relationship satisfaction than capitalization, which is a rather new concept in research. However, the researchers did extra analyses to confirm the results with changing the order of the stages and entering the capitalization responses in stage 2 and accommodation responses in stage 3 and found the same results where Active-constructive capitalization was revealed as the strongest predictor of relationship satisfaction, indicating that this response is the most important predictor of satisfaction.

### The Moderating Effects of Self-Construal on the Relationship Between Perceived Accommodation and Capitalization Responses and Relationship Satisfaction

To test research questions and hypotheses 2, moderation analyses were performed. Firstly, moderation analyses of interdependent self-construal as a moderator for both accommodation and capitalization communicative responses on relationship satisfaction were conducted. As shown in Table 7, the interaction effects between each of the accommodation responses and interdependent self-construal were Active-constructive accommodation [β = −0.06, 95% CI (−0.39, 0.27), *t* = −0.38, *p* = 0.704], Passive-constructive accommodation [β = 0.01, 95% CI (−0.33, 0.36), *t* = 0.08, *p* = 0.938], Active-destructive accommodation [β = 0.21, 95% CI (−0.21, 0.63), *t* = 1.00, *p* = 0.319], and Passive-destructive accommodation [β = 0.05, 95% CI (−0.39, 0.49), *t* = 0.24, *p* = 0.812]. These findings suggested that the relationships between accommodation and relationship satisfaction were not moderated by interdependent self-construal.

**Table 7:** Moderation of interdependent self-construal for accommodation and capitalization reponses and relationship satisfaction.

**Moderator variable**	**Predictor variable**	**β**	**95% CI**	***t***	***p***
Interdependent self-construal	Active-constructive accommodation	−0.06	(−0.39, 0.27)	−0.38	0.704
	Passive-constructive accommodation	0.01	(−0.33, 0.36)	0.08	0.938
	Active-destructive accommodation	0.21	(−0.21, 0.63)	1.00	0.319
	Passive-destructive accommodation	0.05	(−0.39, 0.49)	0.24	0.812
	Active-constructive capitalization	0.26	(−0.41, 0.94)	0.77	0.444
	Passive-constructive capitalization	−0.04	(−0.68, 0.61)	−0.11	0.912
	Active-destructive capitalization	0.49	(−0.13, 1.11)	1.57	0.119
	Passive-destructive capitalization	0.49	(−0.03, 1.10)	1.89	0.062

Similar results were revealed for the capitalization responses. Active-constructive capitalization [β = 0.26, 95% CI (−0.41, 0.94), *t* = 0.77, *p* = 0.444], Passive-constructive capitalization [β = −0.04, 95% CI (−0.68, 0.61), *t* = −0.11, *p* = 0.912], Active-destructive capitalization [β = 0.49, 95% CI (−0.13, 1.11), *t* = 1.57, *p* = 0.119], and Passive-destructive capitalization [β = 0.49, 95% CI (−0.03, 1.10), *t* = 0.1.89, *p* = 0.62] responses showed that interdependent self-construal did not moderate and explain the relationship between these communication responses and relationship satisfaction.

Although not initially planned in the hypotheses, further exploratory moderation analyses were conducted to broaden the understanding of this current sample and results. Intriguingly, in contrast, independent self-construal appeared to moderate the relationship between active-destructive capitalization [β = 0.64, 95% CI (−0.04, 1.24), *t* = 2.12, *p* < 0.05] and passive-destructive capitalization [β = 0.71, 95% CI (−0.19, 1.29), *t* = 2.68, *p* < 0.05] responses with relationship satisfaction. The rest of the accommodation and capitalization responses on relationship satisfaction indicated no moderating effects by independent self-construal (see Table 8).

**Table 8:** Moderation of independent self-construal for accommodation and capitalization reponses and relationship satisfaction.

**Moderator variable**	**Predictor variable**	**β**	**95% CI**	***t***	***p***
Independent self-construal	Active-constructive accommodation	−0.22	(−0.52, 0.08)	−1.44	0.154
	Passive-constructive accommodation	−0.12	(−0.51, 0.26)	−0.62	0.537
	Active-destructive accommodation	0.26	(−0.13, 0.66)	1.31	0.193
	Passive-destructive accommodation	0.38	(−0.04, 0.81)	1.77	0.079
	Active-constructive capitalization	−0.18	(−0.87, 0.51)	−0.52	0.603
	Passive-constructive capitalization	−0.04	(−0.55, 0.63)	0.12	0.904
	Active-destructive capitalization	0.64	(−0.04, 1.24)	2.12	0.036^*^
	Passive-destructive capitalization	0.71	(−0.19, 1.29)	2.68	0.016^*^

* *p < 0.05*.

Hence, the interaction effect of both perceived accommodation and capitalization responses with interdependent self-construal did not moderate and predict relationship satisfaction. The only interacting moderation effects found were between independent self-construal and Active-destructive capitalization and Passive-destructive capitalization responses, which were beyond the scope of this study. Nevertheless, the possibilities of these results will be considered in the discussion section.

## Discussion

Negative communicative behaviors in romantic relationships, such as accommodation in conflicts, have received ample attention in past studies. On the flip side, positive communicative behaviors such as capitalization exchanges toward positive events have been largely overlooked and have not received as much vigor as negative interactional research (Gable et al., 2004, 2012; Smith and Reis, 2012). This current study proposed that it is essential to understand the contemporaneous associations of both negative (accommodation) and positive (capitalization) communicative behaviors to unearth the riddle in predicting positive relationship satisfaction. Prior work has highlighted that couple communication takes diverse forms germane to the cultural context whereupon it occurs and have argued that Western and Asian cultures differ in how they express and perceive beneficial communication behaviors (e.g., Wang et al., 2010; Williamson et al., 2012; Yum et al., 2015; Wang and Lau, 2018). However, positive psychology research studies around the world have been largely based on Caucasian samples and greater diversity is needed in the science of positive psychology (Rao and Donaldson, 2015). Since previous studies were dominated by a Western perspective, exploring both capitalization and accommodation processes may give us a better holistic insight into which communication behaviors are perceived as responsive and fruitful in promoting relationship satisfaction from an Asian viewpoint. In fact, no identified research examining accommodation and capitalization processes has been done in tandem within an Asian context, especially in Malaysia. Therefore, this present study examined the relationship between the two communication processes, (1) accommodation and (2) capitalization, on romantic relationship satisfaction in Malaysian young adults. This study also explored whether interdependent self-construal moderates and explains the differences in communication behaviors, which in turn affects the level of relationship satisfaction.

### Do the Eight Types of Perceived Communication Behaviors in Terms of Accommodation (4 Types) and Capitalization (4 Types) Responses Predict Relationship Satisfaction?

The more well-known piece that literature has appraised in solving the conundrum of relationship satisfaction is accommodation in conflicts. This present study hypothesized that Active-constructive (H1a) and Passive-constructive (H1b) accommodation responses toward the transgressions of a partner in conflicts would positively predict relationship satisfaction, whilst Active-destructive (H1c) and Passive-destructive (H1d) responses would negatively predict relationship satisfaction. Findings revealed that responding in an Active-constructive manner such as discussing problems and altering problematic behavior during conflicts positively predicted relationship satisfaction, supporting H1a. These results align with research done by Rusbult et al. (1991) and Crowley (2006) who established that the willingness to accommodate to the misbehavior of a partner with Active-constructive responses boosts relationship functioning and satisfaction. This indicates that Active-constructive reciprocity will foster satisfaction as it directly engages one partner to be more aware of the maintenance efforts of the other partner in the relationship, which in turn rejuvenates closeness and perceived regard (Overall et al., 2010).

Regarding H1b, it was hypothesized that Passive-constructive accommodation responses during conflicts would positively predict relationship satisfaction; however, the findings did not support H1b. Rather than a significant positive prediction of relationship satisfaction, a slightly negative non-significant result emerged. It was forecasted in this current study that Asians would find forgiving and forgetting the bad behavior of a partner and hoping for things to improve (Passive-constructive) through conflictual circumstances just as beneficial as talking through issues (Active-constructive). This expectation was due to prior work establishing Malaysians and collectivist societies tend to use more indirect and less expressive communication to maintain harmony with others (e.g., Ting-Toomey, 1999; Kennedy, 2002; Oetzel and Ting-Toomey, 2003; Yum, 2004; Bakar et al., 2007, 2014; Wang et al., 2010; Ma-Kellams and Blascovich, 2012). Previous research in Western societies has found unclear and inconsistent results regarding the Passive-constructive accommodation response, making it hard to decipher whether this response is threatening or securing relationship functioning. In their seminal work, Rusbult et al. (1991) and Gable et al. (2004) found the Passive-constructive accommodation response desirable, but later studies by Overall et al. (2010) found the opposite effect. Although not significant, the findings of this current study seem to be more toward the discovery of Overall et al. (2010) because a Passive-constructive accommodation response may be less salient than an Active-constructive response and lead an individual to feel ignored and unappreciated, which could diminish relationship satisfaction. However, further research should continue to investigate the differences of past work as the results in this current study leaned toward a possible negative direction but were indicative of non-significance.

Furthermore, this study found both active and passive destructive accommodation responses detrimental to the relationship satisfaction of an individual. A Passive-destructive response was unmasked as the stronger negative response in predicting satisfaction within this sample in line with H1d. On the contrary, an Active-destructive response appeared to show lower predictive capacity in reducing relationship satisfaction. The rationales of the strength of Passive-destructive accommodation responses are deferred toward the end of the discussion after the capitalization processes are considered as similar results were replicated for capitalization. Despite this, findings still leaned toward the expected direction of both H1c and H1d, exhibiting near to typical findings of the two destructive responses. These results are congruent with past studies that found active and passive destructive reciprocity to the bad behavior of a partner are unaccommodating and lead to dissatisfaction (Rusbult et al., 1991; Crowley, 2006). These findings also denote that not inhibiting destructive impulses would further exacerbate issues, hinder movement toward resolving issues, and lead to distressing relationships (Rusbult et al., 1991; Overall et al., 2010). In short, Active-constructive accommodation responses during conflicts seem to be the most favorable response and allowed dating partners to feel understood, cared for, and validated, which enriched relationship satisfaction. Conversely, Active-destructive, and Passive-destructive accommodation responses seemed unrewarding for dating relationships of Malaysian young adults. The Passive-constructive accommodation responses were the only accommodation results that were not supportive of the hypothesis of this research.

Another complementary yet an understudied communication process in deciphering the relationship satisfaction riddle is capitalization on positive events. Mirroring the hypotheses for accommodation, this study expected that Active-constructive (H1e) and Passive-constructive (H1f) responses to capitalization attempts would positively predict relationship satisfaction whereas Active-destructive (H1g) and Passive-destructive (H1h) responses would negatively predict relationship satisfaction. Consistent with H1e and H1f, results uncovered that providing both enthusiastic support (Active-constructive) and acknowledgment, but an understated support (Passive-constructive) toward the positive events of the partner predicted relationship satisfaction. However, Active-constructiveness had a rather more robust response for satisfying relationships. The findings suggested that the Passive-constructive response stood in contrast to previous research in Western societies as only an Active-constructive response conveyed responsiveness and was associated with higher personal and relationship well-being from the Western lens (Gable et al., 2004, 2006; Pagani et al., 2020). Gable et al. (2006) noted that an Active-constructive response solely highlights the triumphs of the partner and communicates personal significance of the positive event to the discloser. On the other hand, a Passive-constructive response does not convey the same message, which reduces relationship well-being (Gable et al., 2006). Albeit the passive-constructive capitalization responses in this current Malaysian study yielded borderline significance in positively predicting satisfaction, the preliminary correlation demonstrated that this capitalization response had a weak but non-significant relationship to satisfaction. Hence, the results of the passive-constructive capitalization responses should be approached with caution.

Having said that, the results in this current study still appeared to encapsulate the aspect that a Passive-constructive capitalization response such as providing a warm smile and just expressing “*That's nice dear*” toward good news of a partner also tends to be supportive and positive from an Asian perspective. Thus, this finding appears to dovetail nicely with the results of Guo et al. (2018), the only research found in Asia using the perceived capitalization attempts scale of Gable et al. (2004) thus far. They studied familial relationships and demonstrated that both constructive responses of fathers were advantageous for the intrapersonal health and well-being of their children. Therefore, this present research seemed to capture some differences between preferable responses toward personal triumphs of dating couples in Western and Asian countries, echoing the notion that a less overzealous response could still be appropriate and desirable in collectivist cultures (Taylor et al., 2007). According to Choi et al. (2019), Asians also have greater worries about possible repercussions of disclosing personal positive events as it has higher stakes in threatening relational harmony and negative reputations because sharing good news can seem boastful. However, as aforementioned, the findings for the passive-constructive response in this current study narrowly achieved significance. Thus, future research should broaden the sample size and examine other cultural aspects (i.e., harmony values) to better capture the cultural differences for the capitalization process.

Regarding the destructive capitalization reactions, a Passive-destructive response, which is showing disinterest and changing the topic of discussion indicated the strongest predictive value in lowering relationship satisfaction, in tandem with H1h. The same, albeit non-significant trend, was displayed for the active-destructive response (i.e., criticizing and invalidating the partner's good news) leaning toward the anticipated direction of H1g. It can thus be suggested that both destructive responses undermine relationship health as they reject the attempt to develop self-confidence, bids for connection, and engagement of the discloser, which leads to dampening of positive feelings about the event and the relationship (Gable et al., 2004). Repeated destructive responses could also deter the discloser from making capitalization attempts in the future, which may impact the relationship well-being drastically in the long run (Peters et al., 2018).

Furthermore, examining both accommodation and capitalization processes simultaneously provided striking but not surprising results as the most impactful positive response to satisfaction was the Active-constructive capitalization response. This indicates that perceiving that a partner validates the strengths and accomplishments of the discloser in an enthusiastic and encouraging fashion has benefits above and beyond other responses and the accommodation process. These results conform with prior findings that documented support toward positive events was a better predictor of relationship quality than discussions about other circumstances such as adverse events (i.e., Gable et al., 2006, 2012). Thus, as Gable et al. (2006) asserted, “*To put it colloquially, they seem to offer a lot more bang for the buck*” (p. 914). Gable et al. (2006) also found some preliminary evidence that positive event discussions had a greater predictive capacity of couples breaking up at a later point in time. Pertaining to the accommodation process, it can be fairly said that the Active-constructive reaction was also the best response for enriching relationship satisfaction but not as strong as the Active-constructive capitalization response, which seems to be the most salubrious response for dating relationships in Malaysia.

#### Summary and Rationale of Results

Considering the results as a whole, the notion that Asians perceive Active-constructive responses as unbeneficial, uncomfortable, and distressing (Taylor et al., 2007; Wang et al., 2010) is not vouched by this study as this response was found as the most ameliorative reaction for flourishing dating relationships in Malaysia. Therefore, this result supports the universal advantage of Active-constructive responses in both, accommodation, and capitalization processes for relationship maintenance and enhancement.

However, when comparing the two communication processes, there were varying outcomes for the passive-constructive response. This type of communicative behavior seems to have contradicting findings across the literature, and its impact is still not completely clear. While responding in a passive-constructive fashion during capitalization processes was suggestive of beneficial relationship outcomes, accommodating in this manner during conflicts seemed to trend toward being unfruitful for relationship satisfaction in this present research. Even so, due care should be exercised with interpreting these findings. Although the results leaned toward these directions, some results did not appear significant. Be that as it may, it could be argued that the capitalization and accommodation processes are not mutually exclusive, and an effective response depends on the situational context, especially when it comes to Passive-constructive responses. Analogously, a passive-constructive capitalization response to good news of a partner emerged to predict satisfaction positively in this Malaysian sample. In contrast, prior researchers have found that Westerners do not benefit from this type of communication behavior (Gable et al., 2004, 2006; Lambert et al., 2012; Pagani et al., 2020). In light of this, to some extent, this present research unmasked a potential difference in the consequences of the perceived passive-constructive response between Asian and Western countries. On a similar note, scholars have also suggested that perceived responses usually involve a “reality component” (Reis et al., 2004), where researchers should also consider other relationship “realities” such as the expectations of the partner, need for approval, and reasons for disengagement toward the discloser (Gable et al., 2004). Thus, future research should closely inspect these individual differences and motivation to provide a constructive response to understand the complexity of effective communicative behaviors further.

Moreover, concurring with prior research, destructive responses in accommodation and capitalization communication processes were found harmful, regardless of culture. However, one noticeable difference is that the Passive-destructive response in both accommodation and capitalization processes had a greater predictive magnitude in the deterioration of relationship satisfaction when the two processes were examined independently. Active-destructive responses were in the anticipated negative direction but were revealed to be non-significant in predicting relationship satisfaction. On the one hand, it could be that partners of the participants in this study interact in a more vague and evasive destructive way, laying more truth to Asians communicating passively (Guo et al., 2018). On the other hand, the results may indicate that Passive-destructive responses are more detrimental to the receiver than Active-destructive responses (Gottman and Krokoff, 1989). These findings may be because Passive-destructive responses inhibit the ability of a couple to resolve conflicts by “bottling up” emotions, prolonging problems, and causing petty disagreements to escalate out of control, which may cause more long-term problems (Gottman and Krokoff, 1989; Gottman and Levenson, 1992).

Another possible explanation for these results is the stage and status of the relationship of participants in this current study. 73.4% of the sample were in relatively satisfied and happy relationships. Hence, it could be assumed that partners are not reacting in an Active-destructive way, such as showing anger and hostility in conflicts or demeaning and criticizing good news frequently for them to be unsatisfied in their relationships. Besides that, the unintended self-selected bias cannot be ruled out and should be taken into consideration in future research. The results may differ if there was a proportionated balance of participants in the sample who were in satisfying and dissatisfying relationships, which upcoming research needs to obtain and explore.

This research also consists of only dating individuals with an average of 2 years of relationship length. These participants may still be in the “honeymoon” stage of their relationship where trust and intimacy may still be developing, during which they utilize distinctive nature of behaviors such as being more Passive-destructive and more forgiving than couples who have been married for a longer period (Williams, 2012). Passive-destructive responses are possibly more apparent, unnerving, and threatening to premarital or dating couples because partners showing disregard and disinterest may make them feel that their partners are not committed. This relates to a relatively frequent phenomenon in modern dating and a new term called “ghosting,” which shares an overlap with the Passive-destructive response as if an individual “ghosts” another person, they withdraw and avoid the partner entirely (LeFebvre et al., 2019). In other words, when one partner ghosts the other, the immediate consequence is simply an indirect and ambiguous lack of communication (LeFebvre, 2017). As this seems to be a common phenomenon in the current dating atmosphere, it might be another reason why this way of communication had a greater prevalence and strength in declining satisfaction of romantic relationships for this sample. Future research could compare the different phases of relationships and communication behaviors between dating and married couples to understand these results more comprehensively. Despite these rationales, this study suggests a recognition that passive-destructiveness is an unfavorable act to Malaysian dating relationships, maybe more so than active-destructiveness. Further illustrations of the cultural facets, in terms of self-construal and communication are discussed in the subsequent section.

### To What Extend Does Interdependent Self-Construal Moderate the Relationship Between Perceived Capitalization and Accommodation Communication Behaviors on Relationship Satisfaction?

As noted earlier, the present study anticipated Malaysia to be a collectivist country with individuals generally having a dominant interdependent self-construal based on prior studies and was treated as such in hypotheses 2 (Hardin et al., 2004; Amir, 2009; Ting and Ying, 2013). Nevertheless, these current findings did not bear the resemblance of previous discoveries as the interdependence self-construal seemingly did not moderate and explain the differences in both accommodation and capitalization communication behaviors with relationship satisfaction. Thus, these results did not endorse H2a and H2b, suggesting other important factors are at play.

Consequently, given the equivocal findings, further exploratory analyses were conducted though not tested in the hypotheses of this study. It was discovered that independent self-construal instead seemed to moderate the relationships between both perceived active and passive destructive capitalization responses and relationship satisfaction. However, no other significant results were found for accommodation responses. Hence, self-construal appeared to affect the expectations and preferences of perceived responsiveness of sharing good news, just not in the expected direction for Malaysians in this study. The sample in this study also obtained relatively higher mean scores for interdependent and independent self-construal compared to Asian samples in America. These results concur with the findings of Yum (2004) that other self-construal types exist and may explain behavioral variations with greater consistency than the bipolar interdependent-independent self-construal model. One of which is the bicultural self-construal, that is individuals who are high in both interdependent and independent self-construal and are products of modernization and multicultural societies (Kim et al., 1996; Yum, 2004). Given that Malaysia is a melting pot of races, ethnicities, and cultures with unique historical influences (Nagaraj et al., 2015; Park, 2015; The Malaysian Administrative Modernisation Management Planning Unit, 2016), one could speculate that some of the participants in this study could be bicultural. Prior work has asserted that bicultural individuals have rather complex and adaptable identities and are less culturally typical than other self-construal types (Kim et al., 1996; Yum, 2004). They are more flexible and capable of adapting to interactional demands by responding effectively not only to protect their own needs but also the needs of their partner better than other self-construal types. However, this is beyond the scope of this research; thus, future studies could investigate other types of self-construal and whether they moderate the relationship between accommodation and capitalization processes and satisfaction.

Another plausible explanation would be the “modernization hypothesis,” which infers that the behavior of an individual in personal relationships is influenced by the degree of industrialization, westernization, and democratization of the country (Goodwin, 1999). It is also imperative to emphasize that Malaysia was formerly colonized by Western powers such as Britain and, inherently, the Malaysian regime adopted some of the British laws and educational practices which would influence Malaysia's culture (Aziz, 2009; Lee and Low, 2014). Therefore, Malaysians may be affected by social change, modern capitalism and globalization and hold values that are assumed with moving toward individualism, which could explain why the moderating effects of independent self-construal instead of interdependent self-construal were shown in this current study (Goodwin, 1999; Park, 2015). Further, the behavior of an individual could also be influenced by regional subcultures. This current study presumed the national culture of Malaysians to be collectivistic and are governed by interdependent self-construal. However, according to Schmitz (2012), regional sublevels within a country influences the difference in cultural characteristics of various states. This may explain the inconsistency in results of the interdependent and independent self-construals affecting the communication behaviors as participants originated from different states in Malaysia.

Moreover, other scholars have argued that individualist-collectivist stereotypes are becoming meaningless due to other confounding variables such as degree of industrialization, education, and occupation, especially for the younger generations (Kagitçibaşi, 1996; Goodwin, 1999; Matsumoto, 2002; Park, 2015). The sample in this present study represents people of the younger generation who live in urban areas and have relatively high education and economic status. According to Fung (2013), older individuals have a higher tendency to internalize their cultural values compared to younger individuals. Thus, since the participants in this study are of relatively younger ages, there may be a possibility that they have yet to internalize their cultural values (Ho, 2021), explaining the disparities of the interdependent-independent self-construal types. Additionally, Yum et al. (2015) have discovered that although countries are geographically close and in Asia, it does not necessarily mean they have similar values. For instance, Singaporeans were found to adopt traditional and preindustrial beliefs. Conversely, Malaysians reported to hold greater self-expressive and liberal post-industrial beliefs similar to people living in the United States (Yum et al., 2015). Hence, further studies would need to scrutinize the impact of other cultural aspects (e.g., harmony values, and self-expressive values) and widen the range of ages, education, and economic status of participants to truly capture the cultural evolution and communication processes in this modern era.

## Research Implications

Taken together, this current research has highlighted some important implications of romantic relationship communication behaviors, particularly in the realm of dating relationships. This work is the first step toward enhancing our understanding of the accommodation and capitalization processes by looking through an Asian cultural lens. In the overall literature and Malaysian context, there are no identified peer-reviewed studies found investigating the accommodation and capitalization processes and relationship satisfaction simultaneously. Integrating the 2 everyday life communication processes into research provides a better holistic view of how romantic relationships unfold. An active and constructive response was captured as the most salubrious response through capitalization and accommodation processes from a Malaysian standpoint. Prominently, an Active-constructive capitalization response bore the strongest influence on relationship satisfaction above and beyond other responses. A passive and constructive response was suggestive of a positive direction for disclosures of good news but not during conflicts. In contrast, destructive responses in both instances displayed a negative pattern which could steer couples in a downward spiral, irrespective of culture.

The findings of this pioneering study would be a noteworthy addition to literature while contributing to positive psychology theory. This research investigated not only which communication behaviors were beneficial but for whom they are effective for. This study would provide a good comparative data of an Asian country since most studies are conducted and dominated by Western cultures and were subdue to low population and ecological validity as well as pitfalls of research methods. Furthermore, the results could also be a guideline in counseling practices to enhance the “good relationship behavior” and relationship satisfaction of couples by training and cultivating better communication behaviors (Gable et al., 2004), tailored to the preferences and culture of an individual. This knowledge and understanding of human interactional behaviors could be beneficial in designing pre-marital and marriage interventions in Malaysia and other Asian countries. Possible target therapeutic interventions and positive education are fostering constructive communication behaviors, not only to protect relationships with better conflict management but to also enrich romantic relationships by capitalizing on the positive aspects of their lives. Additionally, the findings could serve as a means to educate and create awareness among the general population about good communication practices of which would hope to reduce divorce rates, strengthen relationships and families, and improve the well-being of the society at large, ultimately building flourishing lives.

## Caveats and Future Directions

While this study has made novel contributions to the romantic relationship science literature and goes beyond previous researchers in important ways, future studies should interpret the findings with caution as there were limitations notwithstanding. Firstly, although most of the results were in the expected directions, some of the results appeared non-significant, possibly due to various factors beyond the scope of this research. This study also used self-report data which may induce social-desirability bias; for instance, over-reporting good behavior and higher relationship satisfaction and underreporting undesirable behaviors to be viewed favorably. Moreover, this study focused on the perception of an individual to the response of the partner without directly observing the interaction or investigating how both the discloser and responder feel about the same reactions. Future studies should compare perspectives of both partners and use triangulating methods such as observation in natural settings, in-depth field studies, open-ended surveys, and interviews. Granting all this, it should be noted that responsiveness not only mirrors actual behavior but also the eye of the beholder; therefore, it should be approached with prudence (Reis et al., 2004).

Additionally, women are more attuned to behaviors of partners in daily interactions (Overall et al., 2010). The sample was predominantly female, with 66.9% of females and 33.1% males, making it difficult to accurately compare gender differences. Therefore, future research should assess the complexities of accommodation and capitalization processes in more depth, while examining gender differences. Furthermore, the sample consisted of participants who were relatively young and in dating relationships, of which 73.4% were in satisfying relationships. These participants may also still be in the “honeymoon” relationship phase, during which they utilize and intently focus on different nature of behaviors (Williams, 2012). Responses and perceptions in accommodation and capitalization processes may reveal different patterns in an alternative milieu of long-term, long-distance, distressed, marital, and clinical populations. Future research is recommended to obtain a larger sample size, especially accounting for non-completion of online surveys, to gain a better understanding and generalizability of results.

Lastly, this research was susceptible to ecological fallacy because it assumed that all individuals from a specific culture behave similarly (Freedman, 1999). Thus, some Malaysians may hold bicultural self-construal or more independent than interdependent self-construal. These findings suggest opportunities for future research to investigate other types of self-construal as well as various values that may impact communicative behaviors, namely self-expressive and harmony values. Observing other aspects could valuably aid in capturing the cultural evolution and communication processes in this modern age.

## Conclusion

Conclusively, our need to connect romantically can be ever so fulfilling and enrich our life experiences but can also be arduous and complicated. The contemporaneous associations of both accommodation and capitalization communication processes aids in understanding the complexities of romantic flourishing relationships. This current research has unearthed that attaining relationship satisfaction lies at the heart of responding constructively in romantic relationships as it shows the partner cares and appreciates us. The advantages of active-constructive responses in both relationship processes were more salient and consistent compared to passive-constructive responses. Conversely, in the destructive paradigm, passive-destructive responses emerged as the most unfavorable act in dating relationships in comparison with other destructive responses. This current research has also found that interdependent self-construal did not moderate the communication behaviors. However, captivatingly, unexpected individual and cultural variations were discovered. In the light of these findings, this area of research is essential and further work is necessary to identify additional mediators and effects of these constructs. Regardless, this present research has endowed society one step closer to solving the riddle of achieving relationship satisfaction, and ultimately flourishing romantic relationships. Herein, this study can also serve as a backbone to the knowledge of accommodation and capitalization processes of psychologists and society in Asia and positive psychology literature. Thus, truly strengthening the view of Virginia Satir that “*Communication is to relationships what breath is to life*” (Loeschen, 2017, p. 89).

## Data Availability Statement

The raw data of this research will be made available by the corresponding authors upon request. Further enquiries can be directed to the corresponding authors.

## Ethics Statement

The studies involving human participants were reviewed and approved by Monash University Human Research Ethics Committee (MUHREC, Project Number 10606). The participants provided their written informed consent to participate in this study.

## Author Contributions

All authors contributed to the study and approved it for publication.

## Conflict of Interest

The authors declare that the research was conducted in the absence of any commercial or financial relationships that could be construed as a potential conflict of interest.

## Publisher's Note

All claims expressed in this article are solely those of the authors and do not necessarily represent those of their affiliated organizations, or those of the publisher, the editors and the reviewers. Any product that may be evaluated in this article, or claim that may be made by its manufacturer, is not guaranteed or endorsed by the publisher.
